# Effect of Bioactive and Antimicrobial Nanoparticles on Properties and Applicability of Dental Adhesives

**DOI:** 10.3390/nano12213862

**Published:** 2022-11-01

**Authors:** Marietta Kreutz, Christian Kreutz, Philipp Kanzow, Tobias T. Tauböck, Phoebe Burrer, Christine Noll, Oliver Bader, Bianca Rohland, Annette Wiegand, Marta Rizk

**Affiliations:** 1 Department of Preventive Dentistry, Periodontology and Cariology, University Medical Center Göttingen, 37075 Göttingen, Germany; 2 Department of Conservative and Preventive Dentistry, Center for Dental Medicine, University of Zurich, 8032 Zurich, Switzerland; 3 Institute of Medical Microbiology and Virology, University Medical Center Göttingen, 37075 Göttingen, Germany

**Keywords:** bioactive nanoparticles, antimicrobial nanoparticles, dental adhesive, POSS, SiO_2_@Ag, calcium phosphate, bioactive glass

## Abstract

The aim of the study was to examine the applicability of bioactive and antibacterial nanoparticles to an experimental adhesive. The adhesive (60 wt% BisGMA, 15 wt% TEGDMA, 25 wt% HEMA) was mixed with combinations of 5 wt% methacryl-functionalized polyhedral oligomeric silsesquioxane (MA-POSS) and one kind of bioactive/antibacterial nanoparticles: 1 wt% core-shell silica-silver nanoparticle (SiO_2_@Ag), 1 wt% bioactive glass with bismuth (BAG-Bi) or 1 wt% calcium phosphate (CAP). Pure adhesive served as control. The physicochemical (degree of conversion (DC), linear shrinkage (LS), shear and complex viscosity, water sorption (WS), sol fraction (SF)), biological (antimicrobial effect) and bioactive (mineral precipitation) properties were investigated. DC and LS remained unchanged. The combination of BAG-Bi/MA-POSS resulted in a significantly increased WS and SF compared to control. In addition, the combination of CAP/MA-POSS slightly increased the shear viscosity of the adhesive. The addition of the nanoparticles did not influence the antimicrobial effects compared to the pure adhesive. Improved mineral inducing capacity could be detected in all nanoparticle combinations. The combination of bioactive and/or antibacterial nanoparticles showed improved mineral inducing capacity, but no antibacterial properties. The material properties were not or only slightly affected.

## 1. Introduction

In teeth with deep carious lesions and a symptom-free pulp, the so-called selective caries excavation method allows to keep the microorganisms under a filling to avoid root canal treatment. A compact adhesive composite restoration is then intended to hinder caries progression [1]. However, the level of infection of the pulp cannot be precisely assessed by any clinical examination. For such cases, antibacterial properties of the dental adhesive which is in a direct contact to the microorganisms under the filling might be promising [2,3,4].

A well-known and very effective antibacterial agent is silver, which has been used in medical applications before [5,6]. By reducing the particle size in the nanometer range, it was possible to further improve its reactivity and thus also its antibacterial activity [7]. It has also been shown that the inflammatory response is smaller with nano-sized Ag-particles in comparison with the micro-silver particles [8]. The disadvantage, however, is that the particles tend to form agglomerates and therefore cannot be well integrated into the adhesive matrix [9]. In addition, the ions are released very quickly to the environment, limiting the long-term antibacterial effectiveness [10,11]. For that reason, the silver nanoparticles were coated with silica to achieve a better distribution in the polymer matrix and to achieve a continuous ion release [12,13]. Such antibacterial nanoparticles are promising in an application in dental adhesives.

Beside the bacterial activity in the carious lesions, the parallel degradation of the hybrid layer worsens the prognosis and is still a significant problem despite the continuous improvement of the adhesive systems [14,15]. Another approach is therefore to add ion-releasing (bioactive) particles, e.g. bioactive glass (BAG) or calcium phosphate (CAP) to stabilize the hybrid layer by remineralization [16,17]. When stored in an aqueous medium, such particles release calcium and phosphate ions which are deposited as precipitates on the surface (resin or human tooth) [18,19,20,21]. This mineral precipitation capacity might prevent the hybrid layer from or compensate for the degradation [16,22]. However, it is known that typical bioactive particles tend to aggregate which can lead to worsen material properties, such as higher viscosity [23]. For this reason, their application in dental adhesives is limited. Mineralizing capacity has been also shown by some POSS particles [23,24,25]. Methacryl-functionalized polyhedral oligomer silsesquioxane, MA-POSS, consist of an inorganic silica core and an organic methacrylic side chains [23,26]. Due to the reactive methacrylic groups, the particles are good crosslinkers, disperse better in organic materials and show improved material properties, e.g., degree of conversion or water sorption [23,27,28,29]. In contrast to CAP or BAG-Bi, MA-POSS does not release calcium nor phosphate ions. In MA-POSS, the bioactive effect is probably based on the hydrolyzed unreacted Si-OH groups which serve as nucleation points for ions located in the surrounding solution—from the dental fluid or other bioactive particles [30]. Therefore, a combination of MA-POSS nanoparticles with lower concentration of ion-releasing particles (BAG-Bi, CAP) may lead to an effective bioactivity without strong negative effects on the overall adhesive properties.

In this study, an experimental adhesive was filled by the core-shell MA-POSS nanoparticles combined with the ion-releasing (bioactive) or antibacterial particles to obtain bioactive and antibacterial properties without compromising the applicability of such adhesive. The null hypothesis was that there are no significant differences in the physicochemical properties, but an improved mineral inducing capacity and improved antibacterial effect in the adhesives filled with the nanoparticles.

## 2. Materials and Methods

### 2.1. Experimental Adhesives

An experimental adhesive consisting of 60 wt% BisGMA, 15 wt% TEGDMA and 25 wt% HEMA (Sigma-Aldrich Chemistry, St. Louis, MO, USA) was prepared and magnetically mixed in dark for 10 min as described previously [31]. As photoinitiators, camphorquinone (Sigma-Aldrich Chemistry, St. Louis, MO, USA) and 2(dimethylamino)-ethyl methacrylate (Sigma-Aldrich Chemistry, St. Louis, MO, USA) were added at 0.5 wt% each to enable light-induced polymerization and the mixture was mixed for 10 min with a magnetic stirrer. The samples used for the viscosity experiments were prepared without initiators. 5 wt% of MA-POSS particles (Hybrid Plastics, Hattiesburg, MS, USA) were added and the resin was magnetically mixed again for 10 min. Then, 1 wt% of bioactive or antibacterial particles was added to reach the following experimental groups:

(a) 5 wt% MA-POSS and 1 wt% SiO_2_@Ag core-shell (Nanoshell LLC, Willmington, DE, USA)
(b) 5 wt% MA-POSS and 1 wt% BAG-Bi (ETH Zurich, Zurich, Switzerland)
(c) 5 wt% MA-POSS and 1 wt% CAP (ETH Zurich, Zurich, Switzerland).

The unfilled adhesive served as a control group.

### 2.2. Degree of Conversion (DC)

The DC was investigated by ATR-FTIR (n = 3). The tests were carried out at a wavelength range between 700 cm^−1^ and 4000 cm^−1^ with a resolution of 4 cm^−1^. One test consisted of 26 measurements with an interval of 30 s. For this purpose, one drop of each adhesive group was applied on the diamond crystal to cover the sampling area and polymerized after four initial measurements (120 s) for 60 s (>1000 mW/cm^2^, Bluephase, Ivoclar Vivadent GmbH, Ellwangen, Germany). A background scan was carried out between each test. The benzyl groups were taken as reference and the DC was estimated from the height of the vinyl group peaks before and after polymerization, as described elsewhere [23].

### 2.3. Viscosity

The viscosity was studied by a rheometer (AR-G2 rheometer, TA Instruments, New Castle, DE, USA; n = 3) using a cone plate geometry (40 mm diameter and 2° cone angle). To avoid polymerization, all test samples were prepared without an initiator. Firstly, the linearity was confirmed by an amplitude sweep experiment (γ = 5 to 20% and ꞷ = 20 rad/s). The complex viscosity was measured at the constant deformation of 15% and the frequency range (ꞷ = 10 to 100 rad/s and ꞷ = 100 to 5 rad/s). Two following frequency sweep tests were used to ensure no internal structure or possible polymerization affecting the results. Complex viscosity was estimated at the region where the most systems exhibited a constant viscosity (ꞷ = 30 rad/s). Following, the shear viscosity was estimated through two steady shear tests (10–100 1/s; 100–10 1/s) and the data were fitted by cross-fit model (Newtonian).

### 2.4. Water Sorption (WS) and Sol Fraction (SF)

Five specimens (diameter 6 mm; thickness 2 mm) from each adhesive were prepared in a teflon form and light cured for 60 s from both sides (>1000 mW/cm^2^, Bluephase, Ivoclar Vivadent GmbH, Ellwangen, Germany). Afterwards, the specimens were dried in a desiccator and weighted each two days until the difference between the two following measurements was below or equal 0.1 mg (*m*_0_).

After drying, the diameter and the thickness of the specimens were estimated to determine the volume (*V_m_*_0_). Then the specimens were stored in distilled water at 37 °C and weighted every second day. The cycle was repeated until the difference between two measurements was again below or equal 0.1 mg (*m*_1_). Next, the specimens were dried in a desiccator and weighted again every second day until they reached a steady value (*m*_2_). Based on the determined values, WS and SF could be calculated by the following equations:

(1) $$WS=\frac{\left(m_{1}-m_{2}\right)}{V_{m_{0}}}$$

(2) $$SF=\frac{\left(m_{0}-m_{2}\right)}{V_{m_{0}}}$$

### 2.5. Linear Shrinkage (LS)

The measurements of LS (each group n = 5) were performed by a linometer at room temperature [32]. Twenty microliters of each adhesive were placed on an aluminium plate of the linometer and covered with a 1 mm thick glass plate. Then, the optical fiber of the polymerization light was fixed perpendicular to the specimen surface, and light curing was performed for 60 s. The shrinkage during polymerization results in a vertical movement of the aluminium plate in the direction of the glass plate. This movement was recorded with an infrared sensor at a sampling frequency of 5 Hz for a total of 10 min.

### 2.6. Antibacterial Properties

To prepare tester plates for bacterial adhesion measurement, adhesives (each group n = 6) were applied onto autoclaved glass specimens (Hilgenberg GmbH, Malsfeld, Germany) with a microbrush (Roundtip Applicator, Henry Schein Dental GmbH, Langen, Germany) and polymerized for 60 s (>1000 mW/cm^2^), 24 h before the test. Each test was performed in triplicate. The specimens were shortly cleaned with ethanol and sterile distilled water before embedding in silicone in a 24-well plate (Greiner Bio-One GmbH, Frickenhausen, Germany) and storage at 4 °C.

Inocula of *Streptococcus mutans* (DSMZ-Nr. 20523, German Collection of Microorganisms and Cell Cultures GmbH, Braunschweig, Germany) were prepared from overnight (37 °C, 5% CO_2_) grown agar cultures (Columbia blood agar, Oxoid Deutschland GmbH, Wesel, Germany) in Phosphate-buffered saline (PBS) adjusted to an OD_550_ of 1.0 with a photometer (Ultrospec 1000, Amersham Pharmacia Biotech, Munich, Germany). 1 mL of the bacterial suspension was then applied directly on each test specimen prepared in a well-plate. Bacteria in PBS on a glass specimen was used as negative control. After two hours at 37 °C on a rocking incubator, the bacterial suspensions were aspirated with a pipette and plates were washed with PBS to remove non-adherent cells. To determine the number of living cells an ATP-bioluminescence-assay (ViaLight Plus Assay Kit, Lonza Group Ltd., Basel, Switzerland) was used according to the manufacturer’s specifications.

### 2.7. Mineral Precipitation and Capacity

Cylindrical specimens (diameter 4 mm; thickness 2 mm) were prepared in a teflon form and polymerized for 60 s from both sides (>1000 mW/cm^2^, Bluephase, Ivoclar Vivadent GmbH, Ellwangen, Germany). Artificial dentinal fluid was prepared with accordance to Jungbluth et al. [33] and was slightly modified. It was composed of 1000 mL of distilled water containing 70 g/L albumin (Bovine Serum Albumin Fraction V, AppliChem GmbH, Darmstadt, Germany), 0.193 g KH_2_PO_4_, 0.807 g Na_2_HPO_4_, and 0.617 g CaCl_2_·2H_2_O. Artificial saliva was prepared pursuant to Klimek et al. [34] and again was slightly modified. It consisted of 1.27 g KCl, 0.58 g NaCl, 0.33 g KH_2_PO_4_, 0.34 g Na_2_HPO_4_, 0.225 g CaCl_2_·2H_2_O, 0.16 g NaSCN, 0.16 g NH_4_Cl, 0.2 g urea, 0.03 g glucose, and 0.002 g ascorbic acid mixed into 1000 mL distilled water. Specimens were stored in vertical position for four weeks either in artificial dentinal fluid or in artificial saliva at 37 °C while the medium was changed every second to third day (each group n = 2). Afterwards, the specimens were removed from the media, carefully washed with distilled water and left in a desiccator for drying phase. Then, the surface of the test specimens was sputtered with carbon and examined under the scanning electron microscope (Ultra Plus, Carl Zeiss GmbH, Jena, Germany) at 10 kV. The same specimens were then analyzed by energy-dispersive X-ray spectroscopy (Quanta 200 F, FEI Company, Hillsboro, OR, USA) at 15 kV to estimate the Ca/P ratio.

### 2.8. Statistical Analysis

All statistical analyses were performed using the software IBM SPSS Statistics for Macintosh (version 26, Armonk, NY, USA). Statistical analysis was performed using Kruskal-Wallis-tests followed by multiple pairwise comparisons adjusted to Bonferroni. Pearson correlation was applied to assess the association between oscillatory and steady shear viscosity (*p* < 0.05).

## 3. Results

The results of degree of conversion, shear and complex viscosity, water sorption, sol fraction and linear shrinkage are shown in Table 1.

### 3.1. Degree of Conversion

**Figure 1:**
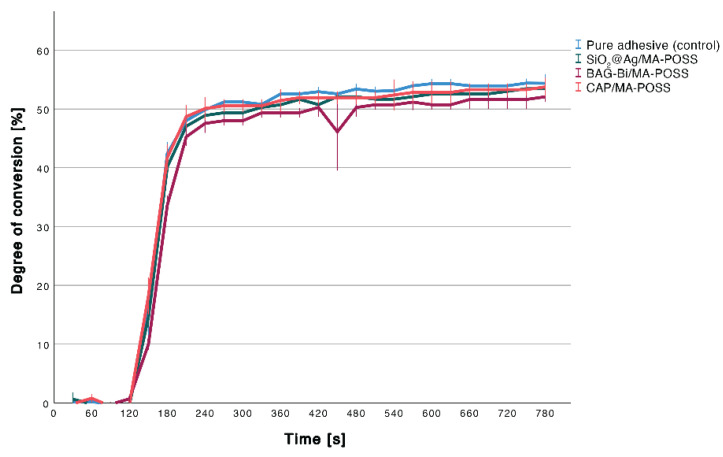
Degree of conversion measured for 13 min. Error bars indicate the standard deviation.

No influence of the nanoparticles on degree of conversion of the experimental adhesive (54.4%) could be observed (*p* = 0.083; Figure 1).

### 3.2. Viscosity

As shown by Pearson correlation (r = 0.939, *p* < 0.001), the complex and steady shear viscosity were associated in a linear way and thus the Cox-Merz rule applies for the studied resins and oscillatory and shear parameters used [35].

**Figure 2:**
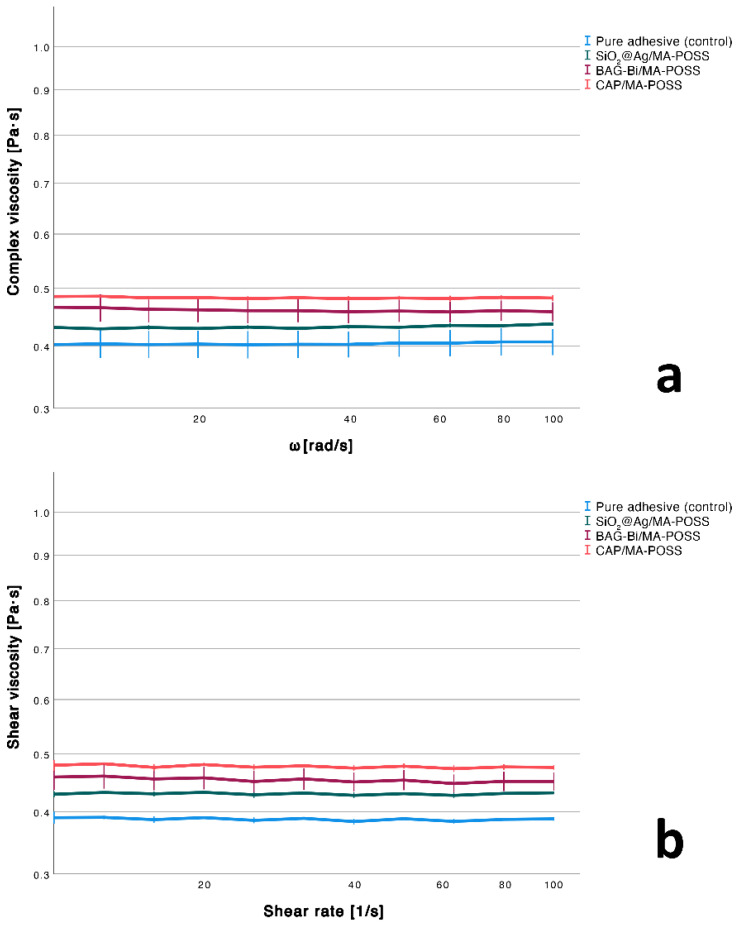
Complex (**a**) and steady shear (**b**) viscosity for different settings. Error bars indicate the standard deviation.

The different combinations of the nanoparticles had a significant effect on shear viscosity (*p* = 0.016) and led to a significant increase in shear viscosity only by the addition of CAP/MA-POSS compared to the pure adhesive (*p* = 0.013). Still, a tendency to viscosity (complex and shear) enhancement, even though not significant, can be seen in all particle-filled groups (Figure 2).

### 3.3. Water Sorption and Sol Fraction

In comparison with the pure adhesive, the sample filled with BAG-Bi/MA-POSS exhibited a significantly higher water sorption (*p* = 0.007) and sol fraction (*p* = 0.005). No significant differences were observed between the other groups.

### 3.4. Linear Shrinkage

**Figure 3:**
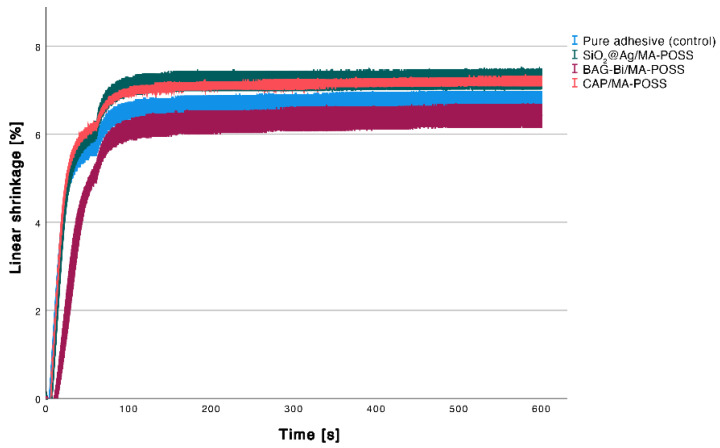
Linear shrinkage measured for 10 min. Error bars indicate the standard deviation.

The Kruskal-Wallis test showed a significant effect of the different combinations of the nanoparticles on linear shrinkage (*p* = 0.002). Compared to control, no significant differences to CAP/MA-POSS (*p* = 0.256), BAG-Bi/MA-POSS (*p* = 1.000) and SiO_2_@Ag/MA-POSS (*p* = 0.149) could be observed (Figure 3). A significantly lower shrinkage was found for BAG-Bi/MA-POSS compared to SiO_2_@Ag/MA-POSS group (*p* = 0.009) and CAP/MA-POSS (*p* = 0.019).

### 3.5. Antibacterial Properties

**Figure 4:**
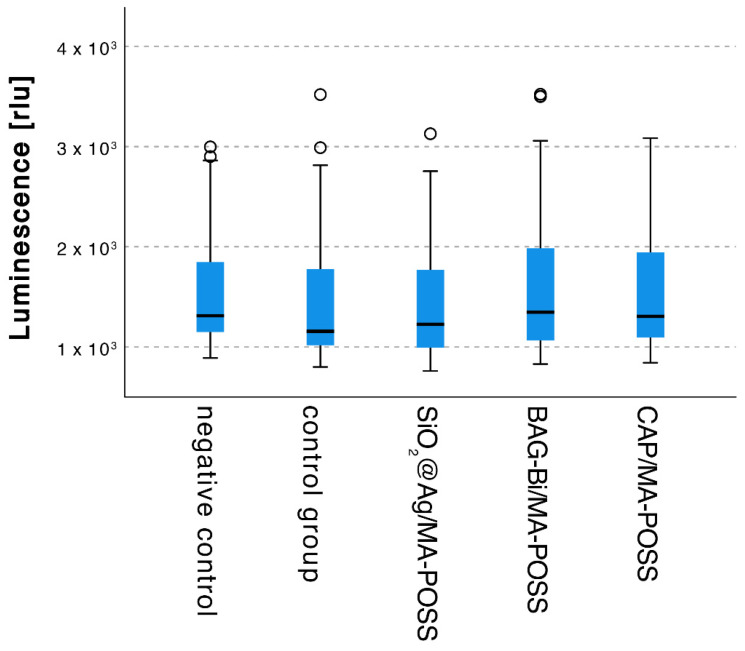
Antibacterial test of the different experimental adhesives. Outliers are marked with a circle (o).

No effect on the bacteria could be observed by the addition of the nanoparticles compared to the control group (Figure 4).

### 3.6. Mineral Precipitation Capacity

**Figure 5:**
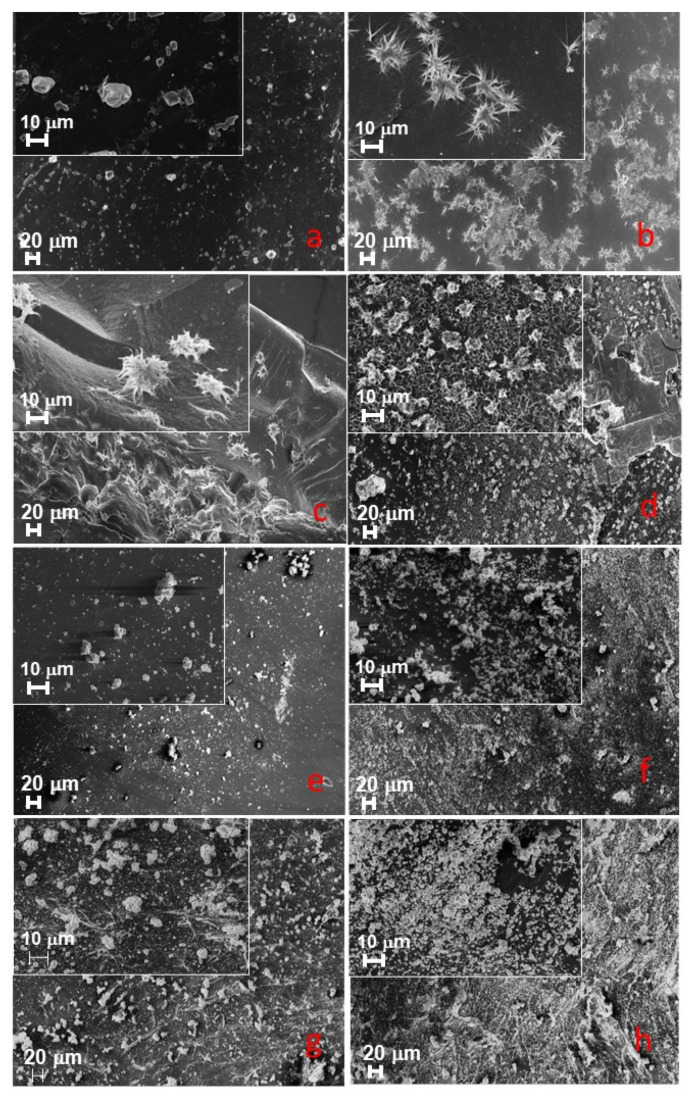
Representative SEM images of the surfaces of the specimens stored in artificial saliva (**a**–**d**) or artificial dentinal fluid (**e**–**h**): (**a**,**e**) control; (**b**,**f**) SiO_2_@Ag/MA-POSS; (**c**,**g**) BAG-Bi/MA-POSS; (**d**,**h**) CAP/MA-POSS.

When specimens were stored in artificial saliva, precipitates could only be detected on the surface of the test specimens (Figure 5b–d), while no precipitates were found on the control specimens (Figure 5a).

After storage in artificial dentinal fluid, mineral precipitates could be observed on all specimens. However, there were visibly more precipitates on the surface of the test specimens with the nanoparticles compared to control (Figure 5e–h).

The molar ratio of Ca/P of the precipitates reached between 1 and 1.2 (artificial dentinal fluid) or 0.4 and 1.0 (artificial saliva), respectively.

## 4. Discussion

The experimental adhesive used in this study was based on the study by Kreutz et al. [31], which analyzed the physicochemical properties and mineral inducing capacity of different experimental methacrylate-based resins containing MA-POSS. The monomers used are the most commonly contained in adhesives or dental resins [36]. The resin with the composition 60 wt% BisGMA, 15 wt% TEGDMA, 25 wt% HEMA and 5 wt% MA-POSS showed the lowest water sorption and a significantly reduced viscosity [31] and was therefore used in this study.

The hypothesis that there is no effect on the physicochemical properties by adding the nanoparticles can be partially accepted. The linear shrinkage was not changed by the addition of any particle combination. The shear viscosity was significantly increased only with the CAP/MA-POSS fillers, solely the BAG-Bi/MA-POSS combination showed a significantly increased water sorption and sol fraction. A low sol fraction has a beneficial effect on biological properties. In the oral environment, monomers and nanoparticles can be released through the saliva and thus enter the body’s circulation. Due to the nanometer size, nanoparticles can be absorbed by cells and lead to inflammation [37]. Similarly, free unreacted monomers could penetrate through the dentinal tubules and cause inflammation of the pulp [38]. For this reason, it is important to keep the amount of the dissolved components on a minimum level.

The increase in water sorption in BAG-Bi group in this study is probably due to hydrophilic and ion-releasing nature of BAG-Bi, as similar results have been observed [18,23]. Since the sol fraction was also increased solely in this group, the possible worse crosslinking in this adhesive could be an additional reason. This is however questionable since the DC measured, although as the lowest, was not significantly different from other groups. In addition, the lowest linear shrinkage after addition of the BAG-Bi particles indicates lower network density in this adhesive. The addition of MA-POSS to an adhesive, on the other hand, led to the opposite effect, in that the more hydrophobic nature of the particles tended to reduce the water sorption [23].

The study results indicate nanoparticles having no significant effect on the degree of conversion. This finding is comparable to those of previous studies presenting no influence on the degree of conversion due to BAG-Bi, MA-POSS or CAP nanoparticles [18,23,26,39]. However, a potentially better conversion may be masked by the apparent conversion which is worsened by the multiple vinyl groups on the MA-POSS particles. Thus, the particles can polymerize with the monomers and improve the strength of the network while the estimated conversion can still be lowered [23]. This phenomenon could explain the slightly lower DC of unfilled adhesive compared to those with MA-POSS particles (Table 1). The effect on monomer conversion has not yet been studied for silver core-shell particles. However, the addition of traditional silver nanoparticles typically leads to a reduction in the degree of conversion even at small amounts [40,41]. This was not observed in this study.

The degree of conversion is strongly associated with the viscosity of the resin blend. Low viscosity typically results in a higher degree of conversion, as the diffusion of the reactive groups is enhanced. Low viscosity is also advantageous for adhesives improving the infiltration into the acid etched collagen matrix. In this study, contrasting the pure adhesive, solely the addition of CAP/MA-POSS significantly influenced the shear viscosity. This may be an indication for a poor particle distribution and a stronger aggregation within the tested adhesive [18,42]. Nevertheless, the slightly higher viscosity did not negatively affect the DC. Although the strong tendency of BAG-Bi to aggregate [19,23], the BAG-Bi/MA-POSS combination only showed a trend to viscosity increase, however, this effect was not significant. This is probably due to the relatively low proportion of added BAG-Bi particles. On the other hand, it is also possible that the addition of MA-POSS has compensated for an increase in viscosity [31]. The lowest viscosity of SiO_2_@Ag group from all filled adhesives indicates again a better particle distribution due to the silica shell. The complex and shear viscosity of all tested groups was unaffected or only increased to an extent that does not hinder the applicability for dentistry [18,43]. Slightly higher values of complex viscosity in comparison to the steady shear viscosity, however within deviation limits, may result from a scarcely higher deformation rate.

The polymerization shrinkage is also related to the degree of conversion [44]. There was also no change in linear polymerization shrinkage due to the combined particles. Although, the MA-POSS alone led to a higher linear shrinkage in some of the studied conventional adhesives [45], it also caused lower polymerization shrinkage and stress in barium borosilicate glass-filled methacrylate composite [46]. The combination of CAP/MA-POSS and SiO_2_@Ag/MA-POSS showed a significantly higher linear shrinkage than the BAG-Bi/MA-POSS combination. This result goes parallel to the observations on WS, SF and DC and supports the assumption about a slightly worsen network density at the BAG-Bi/MA-POSS group in comparison to other filled resins. Similar correlation between DC and polymerization shrinkage was observed on the BAG-filled composite resin by Par et al. [32] and Jäger et al. [47]. The differences could be also due to the possibly different particle distribution in all groups.

An additional mechanical stabilization of the hybrid layer is to be achieved by the addition of nanoparticles that can induce mineral precipitation. After storage in artificial saliva [34] mineral precipitation could be observed on the surface of the specimens filled with nanoparticles, while the pure adhesive showed no crystal growth. The presence of the precipitates on the particle-filled samples is in line with comparable studies which examined the bioactive potential of the individual nanoparticles [18,19,20,23,48]. Besides that, the ion release is faster from the nano-sized particles in comparison to the aggregates and larger particles [19,49]. In the clinical situation the hybrid layer has only limited contact with saliva and is mainly exposed to dentin or dentin liquor. For this reason, the specimens in this study were also stored in artificial dentinal fluid [33] where a precipitation on both surfaces of the test groups and on the control group were observed. However, visibly more precipitates could be seen on the surfaces of the test specimens. Thus, qualitatively all three particle combinations improved or induced the mineral precipitation in both studied media.

There are several factors known to influence bioactivity. In this regard, there are many factors with a crucial role: pH and (super)saturation of the immersion medium and immersion time [34,50], size and concentration of the bioactive particles [19,20,21], as well as the composition and morphology of the adhesive surface [51,52]. Nano-sized particles have shown to exhibit a more efficient ion release and hard tissue regeneration in comparison with micron-sized particles [21,53]. For the long-term immersion, a dissolution of precipitates takes shape. This is followed by a new precipitation. Thus new forms or crystals with different compositions may evolve [52,54]. Conclusively, a regular swap of the immersion medium may affect the growth and composition of the precipitates strongly. For these reasons, a regular immersion medium swap (every other day) as well as the nano-sized particles were chosen for this study.

The initial dissolution of ion-releasing particles within an immersion medium is the first step of the Ca/P crystal precipitation. This leads to an increase of the medium’s (saliva or dentinal fluid) supersaturation, especially around the surface. Followed by the actual precipitation and ion exchange [52].

The precipitates mainly exhibited a cauliflower- or plate-like structure in all particle-filled groups. Such crystals have also been found in other studies [45,55,56] and are described as promising for an application within the field of dental medicine [18,54,55]. This is due to the similarity between the phases and the stochiometric hydroxyapatite, even with lower calcium levels [55]. A longer immersion time may lead to a phase transformation into the crystals with a higher Ca/P ratio. This finding is in accordance with other studies [45,54,55]. However, more experiments with longer immersion times and eventually different ion concentrations are needed to test the formation process of the precipitates on all adhesive groups.

In order to consider new standards of minimally invasive caries therapies, the antibacterial effect was also investigated in this study. No antibacterial effect could be observed in any particle-combination. Although an antibacterial efficacy of SiO_2_@Ag was shown elsewhere [13,57], the concentration of these particles in this study was probably not sufficient for such effect to occur. Another aspect reducing the particle antibacterial activity is that SiO_2_@Ag particles were embedded in a polymer matrix and thus had a small area of contact with bacteria. As a result, the SiO_2_@Ag particles were not sufficiently exposed to the solution and could not be effective. However, a later release of the particles would be beneficial for clinical success, so that studies should be conducted to observe the antibacterial potential over a longer period of time. It was noted that the combination of SiO_2_@Ag and MA-POSS did not have a negative influence on material properties, as observed in other studies for silver particles [41]. As individual nanoparticles, BAG-Bi and CAP were shown to have antibacterial potential [18,19,58,59,60]. However, this is rather based on a release of ions and an associated increase in the pH value and not on direct antibacterial properties like silver particles [60]. Therefore, the antibacterial effect of the ion-releasing particles is time dependent and is the strongest in the initial phase only. Also, in the conditions of reduced ion-releasing ability when embedded in a polymer matrix, this effect may be strongly reduced as seen in our results.

This study showed that while the concentration and combinations of the particles were sufficient for the mineral precipitation inhibition, no antimicrobial effects could be reached in such compositions. Therefore, despite no antimicrobial effect, the studied particle combinations are promising in dental adhesives for the inhibition of a remineralization process.

## 5. Conclusions

The combination of 1 wt% mineral releasing or silica-silver core-shell nanoparticles with 5 wt% MA-POSS in an experimental adhesive showed an improved bioactive potential. However, no additional antibacterial effect was observed for any particle combination. Regarding the studied concentration of the particles, there only was a modest effect on material properties. Therefore, such nanoparticle-combinations were shown to be promising for the application in dentistry.

## Figures and Tables

**Table 1:** Mean values and standard deviation of degree of conversion, viscosity, water sorption, sol fraction and linear shrinkage. Significance differences are marked with different superscript letters.

Experimental Adhesive	Degree of Conversion [%]	Shear Viscosity [mPa·s]	Complex Viscosity [mPa·s]	Water Sorption [µg/mm^3^]	Sol Fraction [µg/mm^3^]	Linear Shrinkage [%]
Pure adhesive (control)	54.4 ± 0.8 ^a^	386.9 ± 2.3 ^b^	402. 4 ± 25.8 ^a^	87.5 ± 6.5 ^b^	0.5 ± 2.8 ^b^	6.8 ± 0.2 ^ab^
SiO_2_@Ag/MA-POSS	53.1 ± 0.4 ^a^	430.1 ± 0.9 ^ab^	432.3 ± 1.4 ^a^	102.5 ± 11.5 ^ab^	8.7 ± 3.7 ^ab^	7.3 ± 0.2 ^b^
BAG-Bi/MA-POSS	51.7 ± 1.4 ^a^	450.8 ± 17.5 ^ab^	459.4 ± 23.4 ^a^	117.1 ± 3.1 ^a^	17.1 ± 6.6 ^a^	6.4 ± 0.3 ^a^
CAP/MA-POSS	53.4 ± 1.2 ^a^	475.7 ± 4.3 ^a^	481.7 ± 4.0 ^a^	91.8 ± 5.7 ^ab^	4.6 ± 6.1 ^ab^	7.2± 0.1 ^b^

## Data Availability

The data presented in this study are available on request from the corresponding author.
